# Thoughts on Trocar Site Hernia Prevention. A Narrative Review

**DOI:** 10.3389/jaws.2022.11034

**Published:** 2022-12-21

**Authors:** A. C. de Beaux, B. East

**Affiliations:** ^1^ Spire Murrayfield Hospital, The University of Edinburgh, Edinburgh, United Kingdom; ^2^ 3rd Department of Surgery, Motol University Hospital, Prague, Czechia

**Keywords:** trocar site, port site, incisional, hernia, prevention

## Abstract

**Background:** Laparoscopic and robot-assisted surgery is now common place, and each trocar site is a potential incisional hernia site. A number of factors increase the risk of trocar site hernia (TSH) at any given trocar site. The aim of this paper is to explore the literature and identify the patients and the trocar sites at risk, which may allow target prevention strategies to minimise TSH.

**Methods:** A pub med literature review was undertaken using the MeSH terms of “trocar” OR “port-site” AND “hernia.” No qualifying criteria were applied to this initial search. All abstracts were reviewed by the two authors to identify papers for full text review to inform this narrative review.

**Results:** 961 abstracts were identified by the search. A reasonable quality systematic review was published in 2012, and 44 additional more recent publications were identified as informative. A number of patient factors, pre-operative, intra-operative and post-operative factors were identified as possibly or likely increasing the risk of TSH. Their careful management alone and more likely in combination may help reduce the incidence of TSH.

**Conclusion:** Clinically symptomatic TSH is uncommon, in relation to the many trocars inserted every day for “keyhole” surgery, although it is a not uncommon hernia to repair in general surgical practice. There are patients inherently at risk of TSH, especially at the umbilical location. It is likely, that a multi-factored approach to surgery, will have a cumulative effect at reducing the overall risk of TSH at any trocar site, including choice of trocar type and size, method of insertion, events during the operation, and decisions around the need for fascial closure and how this is performed following trocar removal.

## Introduction

Laparoscopic and more recently robot-assisted laparoscopic surgery for both benign and malignant conditions of the abdomen has become common place. This was seen from the early 1990’s with the rapid change in practice from open to laparoscopic cholecystectomy (1). Much of the early surgery involved a camera port placed at the umbilicus, and a variety of other ports inserted to allow not only diagnostic but therapeutic interventions. As opposed to one incision, much of laparoscopy involves several small incisions, with each trocar site a possible incisional hernia site. Incisional hernia at a trocar site is often referred to as a “trocar site hernia” (TSH), and it is perhaps better referred to as this rather than a “port site hernia,” as PSH can be confused with the abbreviation for parastomal hernia.

The prevalence of TSH is unclear (2, 3). Imaging such as ultrasound and CT scans appear to diagnose many more TSH than are clinically detectable, and also help clarify the diagnosis when a TSH is clinically suspected (4-7). For example, the TSH incidence in laparoscopic bariatric surgery is usually said to be low single figures of a percent (4). However, in a prospective cohort series with ultrasound follow in a similar study population, one or more of the trocar sites had developed a TSH in 34% of patients (8). This finding has to be set against a follow up CT scan study in a similar study population (the CT scan was done for other reasons but was reviewed for the study). The study included 244 patients, with 732 port sites of 11 or 12 mm diameter, but only 2 fascial defects were identified—all non-palpable, asymptomatic and plugged with fat (9). Clinical versus imaging diagnosis, and the protocol for the imaging, such as with or without Valsalva, may influence detection of TSH. While many small TSHs may have a long natural history of developing into a clinically overt hernia, the explosion in laparoscopic surgery over the last 30 years has not resulted in a similar explosion in the number of TSHs presenting to the surgeon for repair. Indeed, TSH is still a relatively uncommon hernia requiring surgical repair.

Nevertheless, TSHs are evident, and many that present with symptoms of a bulge and/or pain, do require repair, including a small number that present acutely, sometimes within days of the original surgery. Thus, prevention of TSH is likely to be of benefit to patients undergoing laparoscopic surgery. The aim of this narrative review, was to provide an overview of steps along the patient journey that might reduce the risk of TSH. These include possible pre-operative factors, patient risk factors, intra-operative factors as well as post-operative events.

## Methods

A pub med literature review was undertaken on 29 August 2022. The MeSH terms of “trocar” OR “port-site” AND “hernia” was undertaken. There was no attempt at limitation of the search. Papers of any study type including case reports, human and animal research, any language were allowable in the initial search. The title and abstract of the papers from the literature search were scanned by both authors, and possible papers for inclusion selected. Where there was disagreement this was discussed, and generally the abstract included for full text review. Further full text articles were excluded if duplicate information or were not relevant to this review. The focus of this paper was pure TSH, and incisional hernias related to specimen extraction sites, where the trocar site was enlarged, were not included in this review. It was hoped that a reasonable quality systematic or narrative review on TSH in the last 10 years would be identified, and thus limit this review to an update with more recent publications.

A number of topics were considered when reviewing the abstracts and full text papers. These topics were re-operative or patient factors, trocar location, technique of trocar insertion, trocar type, size, length of operation, closure of trocar site and post-operative rehabilitation.

## Results and Discussion

**FIGURE 1:**
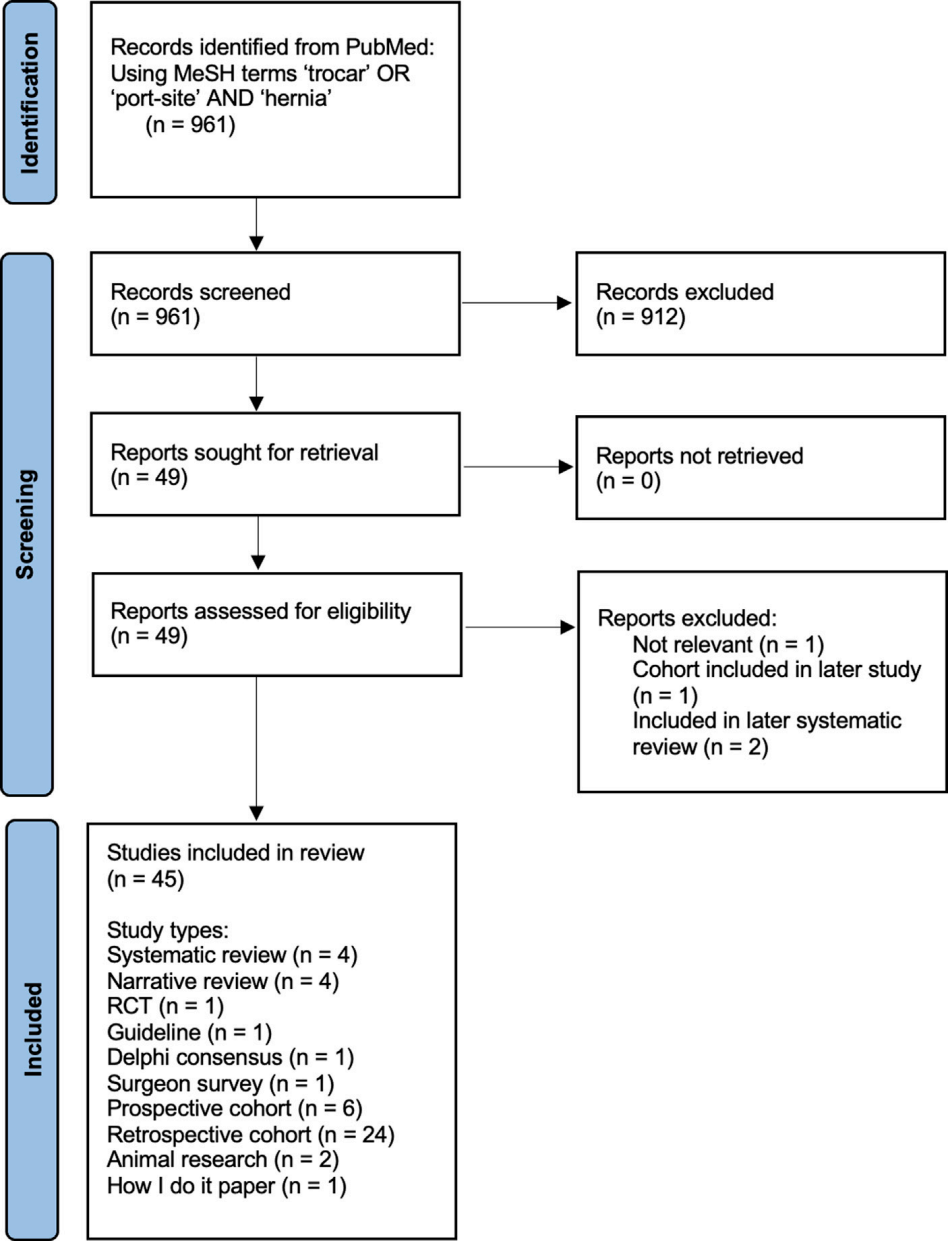
Prisma Flow chart of PubMed Search.

The Prisma flow chart of the publications reviewed is shown in Figure 1. The quality of evidence was generally low, with over half (24 of the 45 publications included) retrospective cohort series. A systematic review published in 2012 was identified (2), and this was used to eliminate studies published prior to this date.

### Pre-Operative or Patient Factors

TSHs are in essence a particular form of incisional hernia. It is not surprising that general risk factors for incisional hernia are also seen for TSH. Namely smoking, obesity, connective tissue disorders, systemic disease such as diabetes mellitus, immunosuppression, the elderly or frail, and sarcopenia, along with rectus diastasis and a history of a previous hernia elsewhere (10-13).

Of interest to this paper on TSH, is the conflicting evidence around pre-existing hernias at the site of the trocar or hernias elsewhere on the abdominal wall. The presence of an umbilical hernia is a risk factor for a TSH at the umbilicus (13, 14). But the water is muddied on this topic by nomenclature. Is the resultant hernia a TSH, a recurrent umbilical hernia or indeed more correctly termed an incisional hernia? And how the umbilical hernia was managed in terms of closure at the end of the operation may also influence the TSH rates. But in a study of umbilical TSH after laparoscopic TAPP inguinal hernia surgery, umbilical TSH was related to the pre-operative presence of an umbilical hernia, rectus abdominis diastasis and surgery for a recurrent inguinal hernia (13).

For many of these patients, pre-habilatation with weight loss, exercise, reducing immunosuppression drugs where possible and reducing the degree of sarcopenia will likely reduce the TSH rate, although evidence that such interventions reduce TSH is lacking in this area. Avoidance of the umbilicus when there is a concomitant umbilical hernia may reduce the TSH rate. Although the patient will still have their umbilical hernia—so it could be argued that in patients with an umbilical hernia, this is the preferred site so that umbilical hernia repair can be incorporated into the surgery.

### Trocar Location Choices: Umbilicus, Midline, off Midline

The umbilicus has been for many laparoscopic operations, the first port insertion site, the usual camera location and often in addition, the specimen extraction location. Given the common co-existence of an umbilical hernia, it is not surprising that the umbilicus seems to be the most common site of TSH (14-16). In a cohort series of laparoscopic bariatric operations undergoing follow up CT scanning, performed prone, the umbilicus was by far the commonest site of TSH (17). However, at the time of writing the study (17), none of the patients identified with a TSH had undergone repair, with nearly all were asymptomatic. Yet in bariatric surgery, most surgeons now would try and avoid the umbilicus, not necessarily purely for TSH prevention, but the fact that the umbilical site is often too far away from the operative field in the left upper quadrant.

Similarly, epigastric trocars, used in the common operation of laparoscopic cholecystectomy, has a TSH incidence muddled by this trocar location often being enlarged for gallbladder extraction (12). But trocars inserted in the midline do seem to have a higher TSH rate, even when closure of the linea alba defect is attempted. The midline insertion is often quick, and the relatively avascular midline does have some advantages over the risk of bleeding when trocars are inserted through the belly of the rectus muscle for example, and especially in the lower half of the abdomen where the inferior epigastric vessels are at risk of injury. In the end of the day, port location is partly determined by the operation being undertaken, but TSH can be reduced if the midline is avoided (18).

### Trocar Insertion Technique: Open v Closed

The open or cut down technique, especially for the first camera port has been promoted as a standard of care at the umbilicus. Again, prevention of a TSH is not the main factor here, rather safe entry to the abdominal cavity. However, open insertion techniques “under vision” are not so easy away from the umbilicus, especially in the obese. While an open cutdown will likely result in a larger “hole” in the abdominal wall, the ability to see the aponeurotic layers at the time of formation and place sutures accurately into these at the beginning of the operation may mitigate against this in terms of risk of TSH. The insertion of sutures at the end of the procedure, particularly when vision down a deep hole and identification especially of the posterior aponeurotic layer, may be less than ideal. A study comparing an open technique versus direct trocar insertion did report a lower incidence of TSH with the direct entry technique (19). Similarly, a study looking at lateral sited trocars reported similar findings (20).

### Trocar Type Bladed/Cutting v Noncutting

Previous reviews have suggested that there is some evidence that cutting blades are associated with a higher TSH risk, compared to more “blunt” or tissues separating trocars (2, 16). Cutting trocars do in general pass more easily through the abdominal wall, but the use of less force during insertion may allow overshoot once the tip of the trocar is in the abdominal cavity. And a cutting tip is more likely to cut through a vessel in the abdominal wall on insertion, rather than push it aside as the trocar is inserted. The pressure effect of the trocar on the tissues may tamponade the vessel until the trocar is removed, and then bleeding commences. So there are reasons to avoid the use of cutting trocars, and they do seem to be less commonly available now.

A porcine animal model has demonstrated that a cutting trocar produces a similar size hole in the fascia compared to tissue separating trocars (21). While that may be true, the effect of cutting the tissues rather than spreading it on insertion, may be compounded over the course of the surgery as the instrument in the trocar is manipulated and forces applied to the abdominal wall.

### Trocar Size: 12, 11, 10, 8, 5 mm

Port size, particularly in the midline does seem to influence TSH. However, TSH are reported even for the 8 mm robot-assisted trocars (22, 23), and indeed, more rarely, in 5 mm trocar sites (24).

Not surprisingly, large single port trocars, again often placed at the umbilicus, are associated with an increase in the TSH as reported in recent systematic reviews (25, 26). However, a number of cohort series have not demonstrated much if any difference in TSH between multi-port and single-port laparoscopic surgery (27).

### Trocar Insertion: Vertical v Angled Towards Operative Field

In general, it is good surgical practice to insert a trocar vertically or perhaps more accurately, perpendicularly to the abdominal wall. Sometimes, a more angled approach, especially where surgery is undertaken in a limited area, such as a laparoscopic cholecystectomy or fundoplication, allows less torque feedback from the abdominal wall, making instrument manipulation through the trocar easier as there is less friction resistance to the instrument in the trocar. However, if the tip of the instrument works sometimes in the upper abdomen, and sometimes in the lower, then as the instrument and thus trocar is manipulated, there can be enlargement of the “hole” with tearing of the tissues of the abdominal wall. So careful insertion, under vision, taking stock of the likely location of surgery within the abdomen, and angling the trocar appropriately, may help reduce the secondary trauma the port may cause during the surgery. However, there is no evidence in the literature to support this common sense approach.

In robot-assisted surgery, an additional element is “port training.” This is a process at the start of surgery, and if the bed position is changed, where each arm of the robotic platform has to be educated about the pivot point or fulcrum—the part of the trocar held by the abdominal wall muscles—around which the robot arms perform their movement. If the pivot point, is not set correctly, then the trocar will pivot around a different set point, potentially causing shearing injury to the abdominal wall, enlarging the defect in the abdominal wall musculature/fascia.

The “Z approach” has been described to trocar insertion, with the location of the hole in the superficial fascia does not quite line up with the deep hole (28). However, no evidence around TSH prevention is presented in this paper to be able to make further comment on this.

### Length of Operation

A number of studies have commented on an increasing TSH rate with increasing length of surgery (2, 24). Again, this is a difficult factor on its own to unravel. Longer operations are likely to be more difficult, involving more manipulation of the instruments and thus the trocars, and result in a more tired or distressed surgeon at the time of trocar site closure. Incorrect port training in robot assisted surgery may also compound this, the longer the operation continues. Which of these factors, if any, contribute to the higher TSH rate, is unknown.

### Closure of the Trocar Site and What Technique?

It is generally accepted that closure of the umbilical trocar site, most midline trocar sites of 10 mm or more, and any port site that is enlarged for specimen extraction (this is a specific trocar site situation and is not discussed further) is good practice (18). An international consensus group had 86.8% agreement that closure of 15 mm ports in all patients was necessary (29). The closure of 10 mm and upwards (and also 8 mm in some robotic operations) trocar sites off the midline is less clear cut, with a broad spectrum of opinion from the “never close” to the “always close” surgeon (29, 30). A retrospective cohort series after laparoscopic sleeve gastrectomy, suggested that closure of the trocar site reduced the TSH incidence by two thirds (31). Another retrospective cohort group in a similar study population reported that closure of the 12 mm epigastric port halved the TSH incidence (32).

A number of closure techniques of trocar sites are described, including direct visualisation and simple suture as a single stitch or a figure of 8 stitch (33). Various needle types, and techniques to pass sutures either blindly or under some vision are reported with good results on short term follow up (34-40). Incorporating haemostats into the suture closure is also described (41, 42). A small series of 15 cases using a “mini-IPOM-plug” reported “good” 6 months outcomes (43). But none of these techniques, including some that have been in use for many years, have gained widespread adoption in surgical practice.

As mentioned above, the umbilicus is a relatively high risk site for TSH. Add in additional factors that makes the patient at higher risk for TSH in general, then perhaps mesh augmentation of the trocar site hernia would be a good idea. One of the few more recent randomised controlled trials, compared prophylactic mesh closure (intra-peritoneal polypropylene omega-3 mesh) versus suture at the umbilicus after laparoscopic cholecystectomy in patients identified as “high-risk” (44). 106 patients were randomised; 92 were included in the final analysis. The TSH rate was reduced in the mesh group to 4.4% compared to 31.9% in the suture group. The wound infection rate was lower in the mesh group (0% v 8.5%), but no other differences between the two groups were noted. The mesh used in this study has now been withdrawn from the market. In a non-randomised study of single port sleeve gastrectomy, both permanent and absorbable mesh reduced the TSH rate at 1 year (45). What mesh, which location of the abdominal wall, what mesh size, in which patient and so on remains unclear from the current literature.

Other techniques for trocar site closure are being considered. A recent study described “controlled heat-induced collagen denaturation” in a living pig model (46). Only 12 trocar sites in 3 pigs were reported on, so more work for sure is required before this potentially enters clinical practice.

### Post-Operative Rehabilitation

There is no literature to help advise specifically on the prevention of TSH after surgery. Return to normal activities of daily living, work and sport is encouraged within the level of discomfort of the patient as is the advice following abdominal surgery in general. Activities that significantly increase intra-abdominal pressure, such as coughing, sneezing and jumping from a height, cannot be influenced to any great degree apart from the latter! Blaming the patient for doing too much too soon, is not an excuse for the resultant TSH.

## Summary Discussion

Clinically symptomatic TSH is uncommon, in relation to the many trocars inserted every day for “keyhole’ surgery. The evidence around prevention of TSH is poor. Much of the literature is retrospective cohort studies and case reports with short term follow up from the original surgery. Prospective cohort series or interventional trials under the rigors of a RCT are few in number.

There will be patients inherently at risk of TSH, especially at the umbilical location, and to a lesser extent the whole of the midline. It is likely, that a multi-factored approach to surgery, will have a cumulative effect at reducing the overall risk of TSH at any trocar site, including choice of trocar type and size, method of insertion, events during the operation, and decisions around the need for fascial closure and how this is performed. Symptomatic TSH appears to be a lot lower than the true TSH rate, which is reassuring. Closure of a trocar site at the end of an operation appears to be surgeon individualised, from the “never closers,” to the “always closers” of 10 mm ports and above. Nevertheless, TSH repair remains a not uncommon elective and emergency hernia operation in view of the volumes of laparoscopic and robot-assisted surgery worldwide.

Future studies should focus on identifying the trocar sites at risk, which is likely to be a combination of patient factors, trocar site, trocar type and so on as mentioned above. This may help identify which trocar sites can be left unclosed, which trocar sites that merit suture closure, and which trocar sites that merit additional mesh augmentation. One of the more difficult areas to examine, which may well be an important element in the risk of TSH, is the surgeon as a risk factor. Knowledge about patients at risk, and training in the operative elements and decision making around trocar type, site, insertion technique and effective closure where necessary tailored to the patient, will likely help reduce the surgeon as an additional risk factor.
